# Plasma Enhanced Atomic Layer Deposition of Plasmonic TiN Ultrathin Films Using TDMATi and NH_3_

**DOI:** 10.3390/ma13051058

**Published:** 2020-02-27

**Authors:** Katherine Hansen, Melissa Cardona, Amartya Dutta, Chen Yang

**Affiliations:** 1 Department of Chemistry, Boston University, Boston, MA 02215, USA; hansen73@bu.edu; 2 Department of Chemistry, Purdue University, West Lafayette, IN 47907, USA; missacardona@gmail.com; 3 Department of Electrical and Computer Engineering, Boston University, Boston, MA 02215, USA; dutta@bu.edu

**Keywords:** atomic layer deposition (ALD), plasmonics, titanium nitride, thin film, optical properties

## Abstract

Transition metal nitrides, like titanium nitride (TiN), are promising alternative plasmonic materials. Here we demonstrate a low temperature plasma-enhanced atomic layer deposition (PE-ALD) of non-stoichiometric TiN_0.71_ on lattice-matched and -mismatched substrates. The TiN was found to be optically metallic for both thick (42 nm) and thin (11 nm) films on MgO and Si <100> substrates, with visible light plasmon resonances in the range of 550–650 nm. We also demonstrate that a hydrogen plasma post-deposition treatment improves the metallic quality of the ultrathin films on both substrates, increasing the ε_1_ slope by 1.3 times on MgO and by 2 times on Si (100), to be similar to that of thicker, more metallic films. In addition, this post-deposition was found to tune the plasmonic properties of the films, resulting in a blue-shift in the plasmon resonance of 44 nm on a silicon substrate and 59 nm on MgO.

## 1. Introduction

While the majority of plasmonics research has focused on noble metals, such as gold and silver, today there is a need to replace these traditional materials with alternatives to make commercially-viable plasmonic devices [1,2,3]. Transition metal nitrides, like titanium nitride (TiN), have proven to be promising due to their real permittivity values comparable to that of traditional metals in the visible range, and tunable optical properties by varying the processing methods and/or variables [4]. Transition metal nitrides possess many promising physical properties, such as high thermal stabilities, high melting points, and compatibility with a wide number of substrate materials. Specifically, transition metal nitrides can be fabricated and integrated more easily into silicon-based devices without the concern of volume expansion and stress at the silicon-plasmonic material interface due to diffusion, as is common with Au due to its low eutectic temperature [5]. Transition metal nitrides are also compatible with complementary metal-oxide-semiconductor (CMOS) technology, enabling lower fabrication costs, easy integration and upscaling in mainstream industrial electronic devices, unlike Au which is a known contaminant to circuitry and, thus, not CMOS compatible [6,7]. TiN demonstrates many of these advantages when compared directly to gold, including thermal stability with a melting point of 2930 °C, compared to gold, which melts at 1064 °C. It also has established feasible methods that are compatible with a wide range of substrates (MgO, alumina, silicon). In addition, when compared to gold, TiN’s plasmonic resonance is located within a similar range, but the resonance can be over a broad wavelength region, which is beneficial for a number of applications [8]. However, high temperatures, either in the form of higher deposition temperatures or post-deposition annealing, is required to achieve metallic TiN, especially with plasmon resonances near 500 nm [4,8,9].

One successful deposition method of producing plasmonic TiN is DC magnetron sputtering [4,8,10]. Particularly, sub-10 nm TiN has been demonstrated on lattice-matched MgO substrates using this method [11,12]. This method often requires temperatures over 500 °C, and TiN optical properties demonstrate a strong substrate dependence when sputtered [8].

Atomic layer deposition has been explored as a powerful method to overcome these setbacks. It has been shown that thermal ALD performed at temperatures from 450 to 600 °C is less sensitive to substrates than sputtering [9]. Xie et al. [13] produced metallic TiN with a growth rate of 0.0178 nm/cycle and resistivity values as low as 300 μΩ cm utilizing titanium (IV) chloride and ammonia precursors. However, such halide-based precursors generally result in clogged gas lines, equipment corrosion, and very low growth rates. Kim et al. [14] compared tetrakis (dimethylamido) titanium (TDMAT) and tetrakis (diethylamido) titanium (TDEAT) as the titanium precursors for thermal ALD of TiN. The resulting TiN films were found to have relatively low carbon contamination, and low resistivities, below 1000 μΩ cm. Films grown using TDEAT as the precursor had lower resistivities but required a higher ALD process temperature and had slower growth rates, 0.1 nm/cycle than those grown with TDMAT, 0.5 nm/cycle [14]. Plasma-enhanced ALD (PE-ALD) can be utilized to lower the temperature of deposition. PE-ALD has been shown previously to produce metallic TiN with metallic optical properties [15,16,17,18]. Musschoot et al. compared a thermal ALD method to a PE-ALD method and was able to produce TiN films with resistivities as low as 150 μΩ cm when using the plasma-enhanced method, versus 53 × 10^3^ μΩ cm for the thermal method. These films were found to have high carbon (9 at%) and oxygen (37 at%) as determined by X-ray photoelectron spectroscopy (XPS) [17]. Otto et al. produced TiN using TDMAT and N_2_/H_2_ as precursors with a growth rate of approximately 0.1 nm/cycle, which was confirmed to be plasmonic from 8–216 nm in thickness utilizing in situ ellipsometry [18]. However, the plasmonic quality of these films after exposure to air upon leaving the ALD chamber was not investigated.

Here, we demonstrate a PE-ALD method with a processing temperature less than 250 °C, which produced plasmonic TiN thin films. The plasmon wavelengths of these films were found to be in the region of 500–600 nm and with low optical loses in that region after air exposure. We performed studies of thickness of 42 and 11 nm on MgO and Si (100). The 42-nm thick films on lattice-matched MgO substrates show a plasmon wavelength of 564 nm with a corresponding ε_2_ of 3.74, and ultrathin films even at thicknesses near 10 nm retain a plasmon resonance. The 11 nm thick films on lattice-matched MgO substrates show a plasmon wavelength of 613 nm with a corresponding ε_2_ of 4.5. Similarly, thick films on Si (100) substrates show a plasmon wavelength of 586 nm with a comparable ε_2_ of 4.21. Importantly, we introduced a hydrogen-plasma post-deposition treatment to further alter the optical properties of TiN while maintaining the thermal budget at the low temperature of 250 °C. We found that such plasma treatments greatly improved the metallic quality of the films, increasing the ε_1_ slope by 1.3 times, from −0.015 to −0.021, on MgO and two times, from −0.012 to −0.024, on Si (100) for 11 nm thick films. Our work demonstrates the feasibility of developing metallic and plasmonic ultrathin TiN films with a PE-ALD deposition and post-deposition treatment that is substrate insensitive and with a low temperature of 250 °C. Our work opens up potentials of investigating a CMOS compatible ultrathin plasmonic material, which have been of interest for flexible transparent optoelectronic devices [19,20] and nonlinear optical applications [21,22].

## 2. Materials and Methods

The TiN films were synthesized in a Gemstar XT plasma enhanced atomic layer deposition system (Arradiance, Littleton, MA, USA). Argon (99.999%, Airgas, Dorchester, MA, USA) is used as a carrier and purging gas. All steps of the TiN film synthesis are carried out in the ALD reaction chamber under vacuum at 250 °C. TiN thin films are grown directly onto the MgO and Si <100> substrates.

Similar to a previously established method to produce metallic TiN [17,23,24], Tetrakis (dimethylamido) titanium(IV) (99%, Strem Chemical, Newburyport, MA, USA), known as TDMATi, is used as the titanium precursor and heated to 65 °C to increase its vapor pressure. TDMATi is exposed to the chamber for 1000 milliseconds, followed by a 10 s purge under 110 sccm argon. The chamber next is exposed to 300 W NH_3_:Ar plasma (10 sccm:100 sccm, respectively) for 20 s, followed by a 10 s purge under 110 sccm argon. This completes one cycle, which is repeated until the desired thickness is reached.

The post-deposition hydrogen plasma treatment on TiN was new. For this step, the substrates are kept inside the ALD chamber at 250 °C. The samples are repeatedly exposed to 5 s intervals of 300 W H_2_ plasma balanced in argon. This is repeated 600 times for a total 50 min exposure to hydrogen plasma.

Structural characterization is performed with Raman spectroscopy (Renishaw, West Dundee, IL, USA) using a 532 nm laser and X-ray photoelectron spectroscopy (XPS) (PHI VersaProbe II, East Chanhassen, MN, USA) using a scanning monochromated Al source (1486.6 eV, 50 W; spot size, 200 µm). X-ray reflectivity (XRR) is performed using a D8 Discover X-Ray System (Bruker, Billerica, MA, USA) with a copper k-alpha source. Thickness is first measured from the raw data using a Fourier transform method. This thickness is used to develop a model using DiffracSuite LEPTOS software, which is fit to the raw data to extract the thickness, roughness of TiN films for various cycle numbers. The density of the TiN films was determined by finding the critical angle (*θ*_c_) from the raw data, (supplementary Figure S1), by finding the angle of half the max intensity. This critical angle was then converted into the film density, ρ, using Equation (1) [25]:

(1) $$\rho =\;\frac{\theta_{c}^{2}\pi A}{r_{e}\lambda^{2}ZN_{A}}$$

where A is the mass number, r_e_ is the classical electron radius (2.82 × 10^−15^ m), λ is the X-ray wavelength (1.5418 Å), Z is the atomic number, and N_A_ is Avogadro’s number. The A and Z terms were determined based on the composition of the film found by XPS.

Characterization of optical constants is performed using variable angle spectroscopic ellipsometry (J. A. Woollam Co. Inc. V-VASE, Lincoln, NE, USA) from wavelengths of 400 to 1200 nm and angles of 65°, 70°, and 75°. This data is modeled with a Drude-Lorentz function with one Lorentz operator, as demonstrated previously over this wavelength range [12], to obtain the real (ε_1_) and imaginary (ε_2_) values of permittivity.

## 3. Results

**Figure 1:**
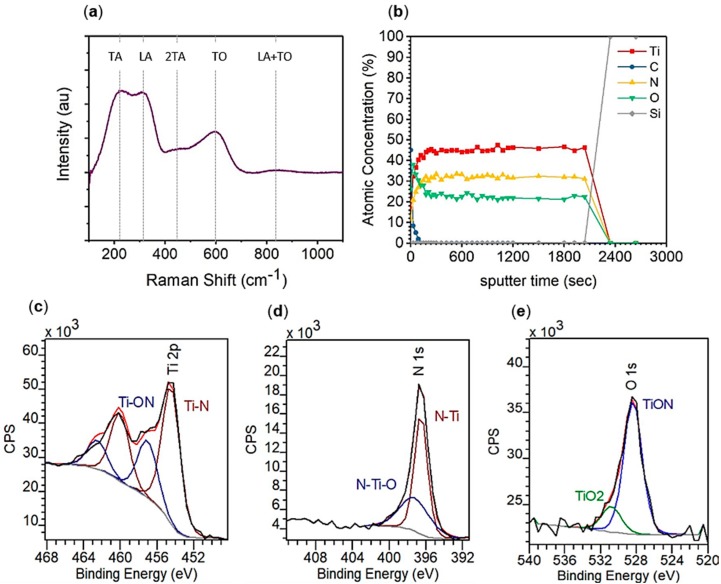
Structural characterization of 400 ALD cycles TiN on MgO using (**a**) Raman spectroscopy and XPS (**b**) depth profile, (**c**) titanium 2p region, (**d**) nitrogen 1s region, and (**e**) oxygen 1s region.

To confirm successful synthesis of a titanium nitride thin film from TDMATi and NH_3_ plasma at 250 °C, structural characterization was first carried out (Figure 1). Raman spectroscopy, performed on a film grown by 400 ALD cycles on an MgO substrate (Figure 1a), confirms TiN. We observed the three broad peaks associated with first order Raman scattering in titanium nitride [24]. Two low-frequency peaks, 220 (transverse acoustic, TA) and 310 (longitudinal acoustic, LA) cm^−1^, are caused by acoustical phonons from the titanium ions. The higher frequency peak, 580 cm^−1^ (transverse optic, TO), is from optical phonons from the lighter nitrogen ions. We additionally observed a secondary Raman scattering peak at 450 cm^−1^ (2TA) and a two-phonon scattering mode at 825 cm^−1^ (LA + TO) also consistent with TiN. [26,27]. The ratio of the optical and acoustic peaks indicates non-stoichiometry within the TiN film. As the TO mode is approximately two thirds the intensity of the acoustic modes, it is concluded that the TiN produced by TDMATi and NH_3_ plasma is nitrogen deficient (TiN_1-*x*_).

XPS was employed for more quantitative characterization of TiN films grown by 400 ALD cycles on a silicon substrate. A depth profile (Figure 1b) of the TiN film shows the film composition on average to be 45 at% titanium, 32 at% nitrogen, 24 at% oxygen, and 0 at% carbon within the bulk of the film. Thus, the Ti:N ratio throughout the film is approximately 1:0.71, consistent with the conclusion obtained from Raman Spectroscopy that that the TiN is nitrogen deficient. The titanium 2p peak region (Figure 1c) has resolved spin-orbit components, 2p_1/2_ and 2p_3/2_, in which the binding energy of the 2p_3/2_ peak is utilized to indicate the chemical state. The titanium 2p_3/2_ peak can be resolved into two peaks, one located at 454.4 eV which is indicative of Ti–N and the other located at 456.9 eV indicative of Ti–ON which is consistent with metallic TiN. The nitrogen 1s peak region (Figure 1d) has a peak that can also be resolved into two peaks, one located at 396.5 eV which is indicative of N–Ti and the other located at 397.5 eV indicative of N–Ti–O. The ratio of the Ti–N:Ti–ON and N–Ti:N–Ti–O peaks both indicate the film is 69% TiN and 31% TiON.

There is a non-negligible oxygen content in the TiN film, 24 at%. The oxygen origin is attributed to the impurity in the precursor TDMATi [28] and the relatively high base pressure of 100 mTorr present in the benchtop ALD system d. We note that the oxygen content is sufficiently low, lower than that reported (37 at%) which still resulted in low resistivity films 150 μΩ cm [17], and that such oxygen content is not expected to prevent metallic optical behavior. Additionally, the oxygen 1s peak region (Figure 1e) has a peak that can be resolved into two peaks, one located at 528.4 eV indicative of TiON and another at 530.7 eV indicative of TiO_2_. The presence of oxygen in a TiO_2_ state has been proposed to be better for the metallic properties of the film due to better conductivity than TiON.

**Figure 2:**
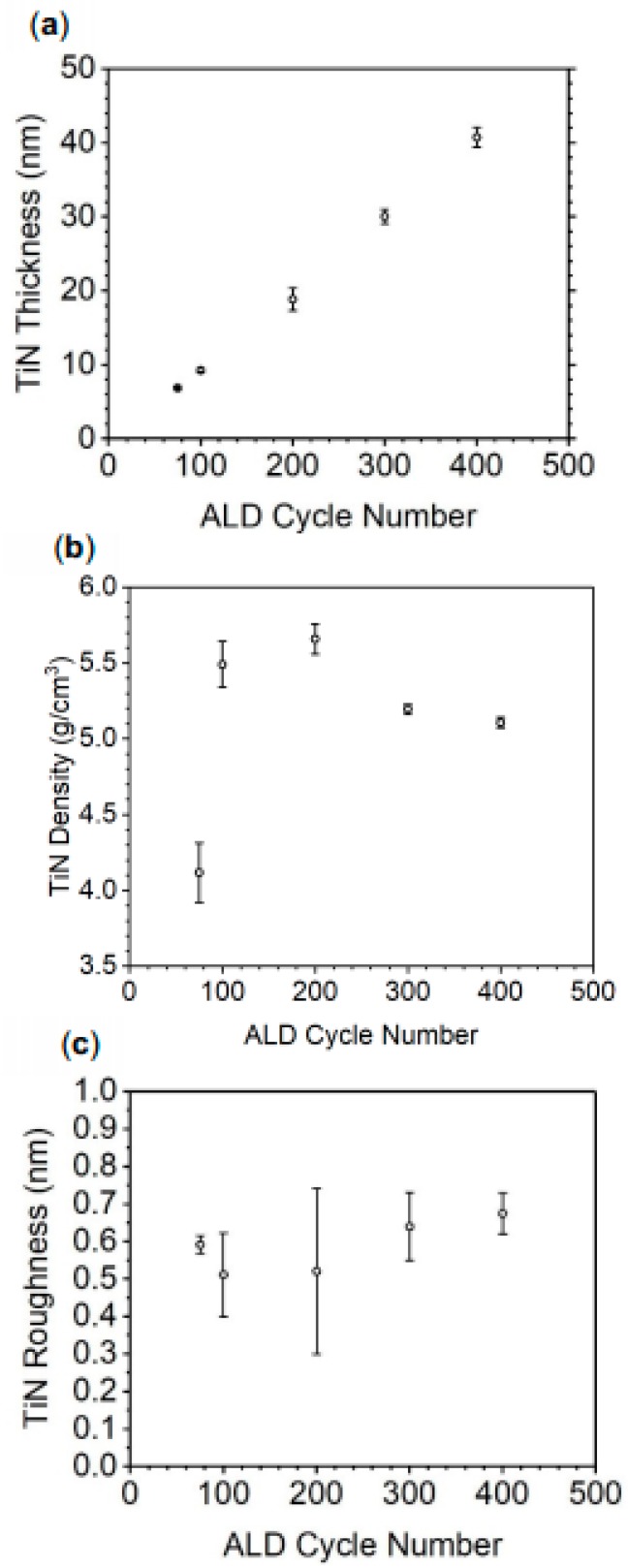
TiN thin film properties from XRR: (**a**) thickness, (**b**) density, and (**c**) roughness as a function of the number of ALD cycles.

Atomic layer deposition was performed by repeating exposures of TDMATi and NH_3_ plasma, which makes up one cycle and the growth rate is expected to be linear over cycle number. To confirm this behavior and determine the TiN growth rate, density, and surface roughness of the TiN, X-Ray Reflectometry (XRR) (Figure 2) was employed to analyze TiN thin films deposited on silicon (100). As observed in Figure 2a, the growth rate is 0.097 ± 0.004 nm per cycle (or approx 9.7 nm per 100 cycles). TiN film density (Figure 2b) approaches the bulk density of TiN, 5.21 g/cm^3^, as film thickness increases. The TiN thin film prepared by 100 ALD cycles has an average density of 4.11 g/cm^3^ while the TiN prepared by 300 and 400 cycles has a density of 5.19 and 5.11 g/cm^3^, respectively. XRR measurements show that the TiN roughness does not vary significantly as a function of thickness (Figure 2c). On average, TiN thin films on silicon have an average roughness of 0.58 nm, indicating that the films are smooth over the substrate surface.

**Figure 3:**
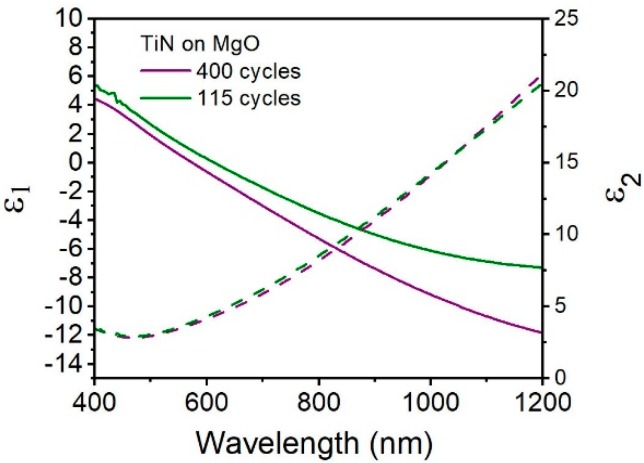
Real (solid line) and imaginary (dashed line) values of permittivity (optical constants) for TiN on MgO, TiN film thickness are 42 (purple) and 11 (green) nm, prepared by 400 cycles and 115 cycles.

We then characterized the optical properties of these TiN thin films and evaluate their potential to be used in plasmonic devices. The optical properties of the as-prepared TiN were measured using spectroscopic ellipsometry (SE). As seen in Figure 3, TiN films on magnesium oxide (MgO) substrates with thickness of 42 and 11 nm were measured. MgO was selected as the initial test substrate due to its near-perfect lattice match with TiN: both are cubic crystal systems with lattice constants of 4.23 Å for TiN and 4.21 Å for MgO. SE using a Drude-Lorentz function with one Lorentz operator [12] reveals the real and imaginary parts of the dielectric function (ε_1_ and ε_2_) over the range 400–1200 nm. First, we observed that the ε_2_ values for the TiN films are similar for both thicknesses, displaying values over wavelength consistent with previous findings [2,18], indicating that the TiN films has similar optical losses. For TiN thin and ultrathin films, ε_2_ reaches a minimum value of approximately 2.9 at the wavelength 465 nm. More importantly, it is observed that the slope of ε_1_ is negative for both TiN samples, indicating the TiN is optically metallic. As expected based on previous studies [18], thicker TiN films are more metallic, especially in the higher wavelength region of 800–1200 nm. The plasmon wavelength, defined as ε_1_(λ) = 0, is located at 574 nm for TiN prepared by 400 cycles. The ultrathin film shows a comparatively red-shifted plasmon resonance of 613 nm, which has been seen previously in ultrathin TiN [12,18]. This demonstrates that TiN of various thicknesses produced by this PE-ALD method has tunable, visible light plasmon resonances for thin films.

Unique from traditional plasmonic metals, TiN has variable optical properties in the near-IR and visible regions. One method of tuning the optical properties is through high-temperature annealing in vacuum, which results in a more metallic film, a down-shift in ε_1_ and may reduce ε_2_ [3,29,30]. However, high-temperature anneals are not ideal for all applications and, therefore, having a low-temperature post-deposition treatment to achieve varied optical properties is advantageous. One alternative to high-temperature annealing is plasma post-treatment, which has been demonstrated previously with ALD metal films to modify the surface and decrease resistance [29]. Yun et al. [30] report a H_2_/N_2_ plasma post-deposition treatment that reduced surface oxygen contamination as well as carbon contamination throughout ALD TiN films that had been exposed to air for 30 days. Such post-deposition plasma treatments were found to reduce the resistivity of TiN films from 25000 μΩ cm to 3000 μΩ cm with an H_2_/N_2_ plasma treatment [30] and to reduce increase in resistivity due to air exposure using a H_2_ plasma treatment [31]. Our work aims at investigation of the effect of the hydrogen plasma treatment on the optical properties of ultrathin TiN thin films (11 nm). This hydrogen treatment occurs at the same temperature as the TiN deposition, 250 °C, using a 300 W H_2_ plasma balanced in argon, repeating a five second exposures to the plasma for a total of 50 min exposure time.

Compositional analysis by XPS of a post-deposition hydrogen plasma treated sample (supplemental Figure S2) shows the following key features. First, the titanium 2p_3/2_ peak (Figure S2a) and the nitrogen 1s peak region (Figure S2b) can still be resolved into the two peaks indicative of TiN and TiON with the ratio of 69:31. The elemental ratio after the post-deposition plasma treatment remains 44:32:24 Ti:N:O. Second, there is a notable change in the chemistry of the oxygen species in the film (Figure S2c), with the loss of the peak located at 528.4 eV indicative of TiON and a new peak located at 533.4 eV which accounts for ~13% of the oxygen content in the film. Such a peak is indicative of four possible chemistries: 1) SiO_2_ which has an O 1s peak at 532.9 eV, 2) organic C–O which has an O 1s peak at ~533 eV, and 3) a metal hydroxide (M–OH) or non-lattice, weakly adsorbed oxygen species which can be found from the range of ~533 eV. The absence of the silicon 2p peak and the carbon 1s peak indicate (Figure S2d) there is no notable silicon or carbon in the film. Therefore, the 533.4 eV peak is likely indicative of a metal hydroxide or non-lattice, weakly adsorbed oxygen species. Typically, common compositional characterization tools, such XPS and EDX, cannot provide content information on hydrogen. In the very limited hydrogen content studies of the ALD-grown nitride, elemental profiling by elastic recoil detection analysis (ERDA) has been used. For example, Bommali et al. [32] reported an increase in hydrogen content in α-SiN_x_:H films after a one-hour hydrogen plasma treatment from 8 ± 2 atoms/nm^3^ to 14 ± 2 atoms/nm. We expect a similar increase in the hydrogen content of our films after the post-treatment.

**Figure 4:**
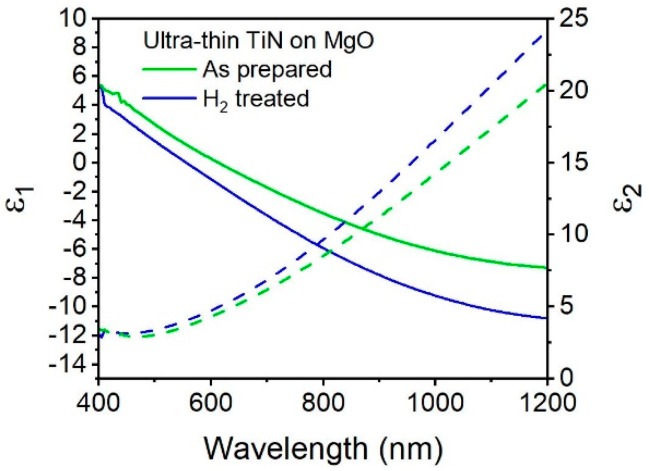
Real (solid lines) and imaginary (dashed lines) values of permittivity of ultrathin TiN (100 cycles) on MgO as prepared (green) and after hydrogen plasma anneal (blue).

To observe how the post deposition hydrogen plasma treatment affects the optical properties, we plot the dielectric functions of as-prepared TiN and H_2_ plasma treated TiN (Figure 4). The hydrogen plasma treated TiN (blue) has a plasmon resonance of 554 nm, a 59 nm blue-shift compared to the as-prepared sample. For comparison, it has previously been shown that the plasmon resonance of sputtered TiN films can be been blue shifted by 45 nm after annealing at 600 °C and 71 nm at temperatures of 700 °C [4] Previous ALD syntheses have demonstrated plasmon wavelength blue shifts of 52 nm after post-deposition annealing at 600 °C [9], and 175 nm after post-deposition annealing at 900 °C [3]. The effect of a post deposition hydrogen plasma on the plasmon wavelength can be similar to that of a high temperature anneal, where an increase in the carrier density results in a decrease in ε_1_ and, thus, a blue-shift in the plasmon resonance [12]. An increase in carrier density is also known to result in an increase in ε_2_ [33], in accordance with our observations.

The ε_2_ values are higher in the hydrogen treated sample: for instance, at a wavelength of 800 nm the value of ε_2_ is 9.7 in the hydrogen treated sample and 8.2 in the as-prepared sample, and this difference only increases as the wavelength proceeds toward the infrared region. However, the values of ε_2_ are similar at their respective plasmon wavelength. For hydrogen plasma-treated TiN, the value of ε_2_ at the plasmon wavelength is 4.04, while the as-prepared sample has an ε_2_ value of 4.5. Figure 4 shows that TiN treated with hydrogen plasma (blue) results in an increase in the metallic behavior of the thin film, as seen by a 1.3 times increase in the slope of ɛ_1_ over wavelengths. Re(ε) for the plasma-treated TiN reaches a value of −9.1 at 1000 nm, significantly lower than the as-prepared TiN, which as a value of −6.1 at 1000 nm, and similar to that of the thicker TiN (42 nm) which reaches a value of −9.2 at 1000 nm. Therefore, taking both parts of the dielectric function into consideration, post deposition treatment with hydrogen plasma is an effective way to tune the optical properties of ultrathin plasmonic TiN on MgO while making the film more metallic.

**Figure 5:**
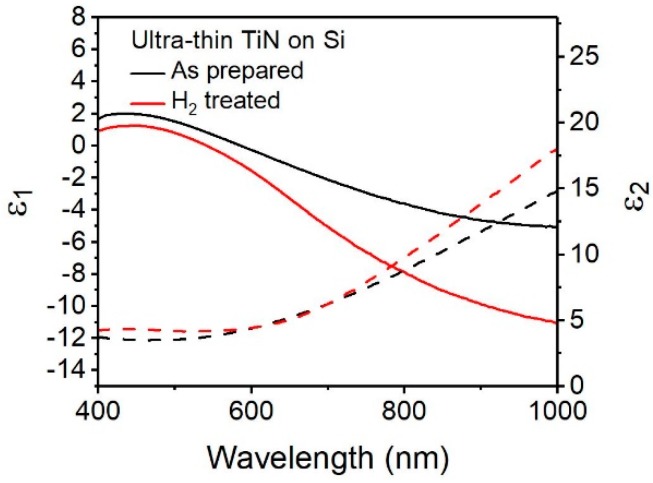
Real (solid line) and imaginary (dashed line) values of permittivity for ultra-thin TiN on Si <100> substrate as prepared (black) and after hydrogen plasma treatment (red).

TiN’s compatibility with silicon is another advantage of alternative plasmonic materials over gold or silver. However, unlike those traditional plasmonic metals, TiN has been demonstrated to have substrate-dependent optical properties and, therefore, it cannot be assumed that the optical properties of TiN on one substrate will be identical to another [8,9,34]. Figure 5 shows the as-prepared TiN (black) has plasmonic character on Si <100> for near 10 nm TiN. The slope of ε_1_ in the visible region is similar to ultrathin TiN on MgO in the region of 500–900 nm, though the TiN shows dielectric behavior (a flattening of ɛ_1_) at wavelengths over 900 nm. This is, in part, attributed to the oxygen composition in the TiN, and is consistent with previous findings of TiN thin films on silicon with oxygen contents between 10% and 25% [10,35]. With the post-deposition plasma treatment this flattening of ɛ_1_ is reduced, consistent with Yun et al. findings that such treatments reduce surface oxidation [30] The slope and position of ɛ_1_ indicate a broad plasmon resonance centered at 586 nm, with a corresponding ɛ_2_ value of 4.21. That the plasmon wavelength is red-shifted for TiN on Si compared to the plasmon wavelength of TiN on MgO is likely due to lattice mismatch between silicon and TiN [9]. The trends observed for $\;\varepsilon_{1}$ and $\varepsilon_{2}\;$, post-hydrogen treatment, are similar to the ones observed before, and can be explained in a similar manner as above. Therefore, we demonstrate that plasmonic TiN can be deposited on Si via PE-ALD and that post-deposition treatment with hydrogen plasma is a viable method to tune the optical properties of prepared TiN on silicon and result in a metallic film.

## 4. Discussion

In this work, we have demonstrated ultrathin plasmonic TiN that was synthesized by PE-ALD using TDMATi and NH_3_ plasma precursors at 250 °C followed with a post-deposition hydrogen-plasma treatment at 250 °C. XPS and Raman spectroscopy indicated that the TiN is non-stoichiometric TiN_0.71_, with no carbon contamination. XRR indicated a growth rate of 0.097 ± 0.004 nm/cycle with an average roughness of 0.58 nm, indicating that the films are smooth over the substrate surface. TiN is found be optically metallic for both thick (40 nm) and ultrathin (11 nm) films on MgO and Si <100> substrates, with visible light plasmon resonances in the range of 550–650 nm with low optical losses (Table 1). Additionally, we show that the plasmonic properties of the film can be tuned using a low temperature hydrogen plasma post-deposition treatment, resulting in a blue-shift in the plasmon resonance of 44 nm, from 586 to 542 nm, on a silicon substrate and 59 nm, from 613 to 554 nm, on MgO. Importantly, we also show that such post-deposition treatments greatly improve the metallic properties of the ultrathin films (11 nm), increasing the ε_1_ slope by 1.3 times, from −0.015 to −0.021, on MgO and 2 times, from −0.012 to −0.024, on Si (100), to behave just as metallic as thicker samples, −0.022 of 42 nm on MgO. Therefore, we have demonstrated a low-temperature PE-ALD deposition and post-deposition treatment of ultrathin plasmonic TiN that is substrate insensitive.

Ultrathin plasmonic materials are advantageous for their use as transparent and flexible electrodes in optoelectronic devices, and key to the development of bendable and wearable systems [19,20], and ultrathin films with thicknesses approaching a few monolayers result in strong confinement that can result in quantum effects, such as nonlinearity [21]. Our work opens up potentials of investigating a CMOS compatible ultrathin plasmonic material for these applications.

## Figures and Tables

**Table 1:** Summary of plasmon resonances and ɛ_2_ values for PE-ALD TiN on MgO and Si substrates.

Film Thickness(nm)	Substrate	Plasma Anneal	λps (nm)	ɛ_2_	ɛ_1_ Slope
42	MgO	no	574	3.74	−0.022
11	MgO	no	613	4.50	–0.015
11	MgO	yes	554	4.04	–0.021
11	Si	no	586	4.21	–0.012
11	Si	yes	542	4.20	–0.024

## Supplementary Materials

The following are available online at https://www.mdpi.com/1996-1944/13/5/1058/s1, Figure S1: Raw XRR data of TiN films on Si (100) with varying the cycle number, Figure S2: Structural characterization of 100 ALD cycles TiN on Si after H_2_ post-deposition treatment using XPS.
