# Supplementary material for: Plasma Enhanced Atomic Layer Deposition of Plasmonic TiN Ultrathin Films Using TDMATi and NH3
Source: Materials (Basel). 2020 Feb 27;13(5):1058. doi: 10.3390/ma13051058 (PMC7084610; doi:10.3390/ma13051058)
Supplement: Supplementary file 1 [file materials-13-01058-s001.pdf]

materials

Supplementary Materials

Plasma Enhanced Atomic Layer Deposition of Plasmonic TiN Ultrathin Films Using TDMATi and NH_3

Katherine Hansen [1,†], Melissa Cardona [2,3,†], Amartya Dutta[3], and Chen Yang [1,3,*]

[1] Department of Chemistry, Boston University, Boston, MA 02215, USA; hansen73@bu.edu
[2] Department of Chemistry, Purdue University, West Lafayette, IN 47907, USA; missacardona@gmail.com
[3] Department of Electrical and Computer Engineering, Boston University, Boston, MA 02215, USA; dutta@bu.edu
* Correspondence: cheyang@bu.edu
† These authors contributed equally to this work.

Received: 27 December 2019; Accepted: 20 February 2020; Published: date

Supplementary Information

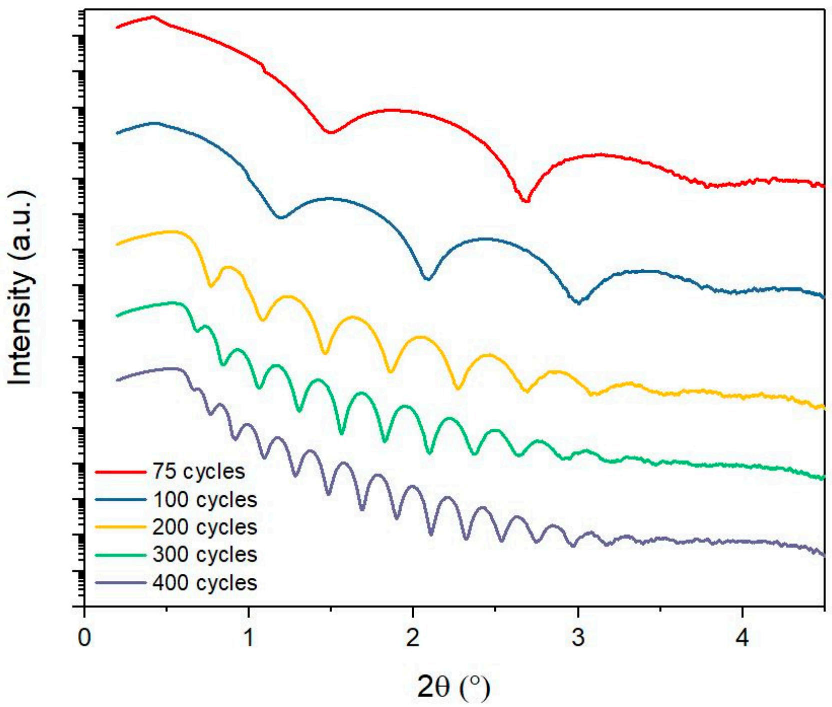

Figure S1. Raw XRR data of TiN films on Si (100) with varying cycle number.

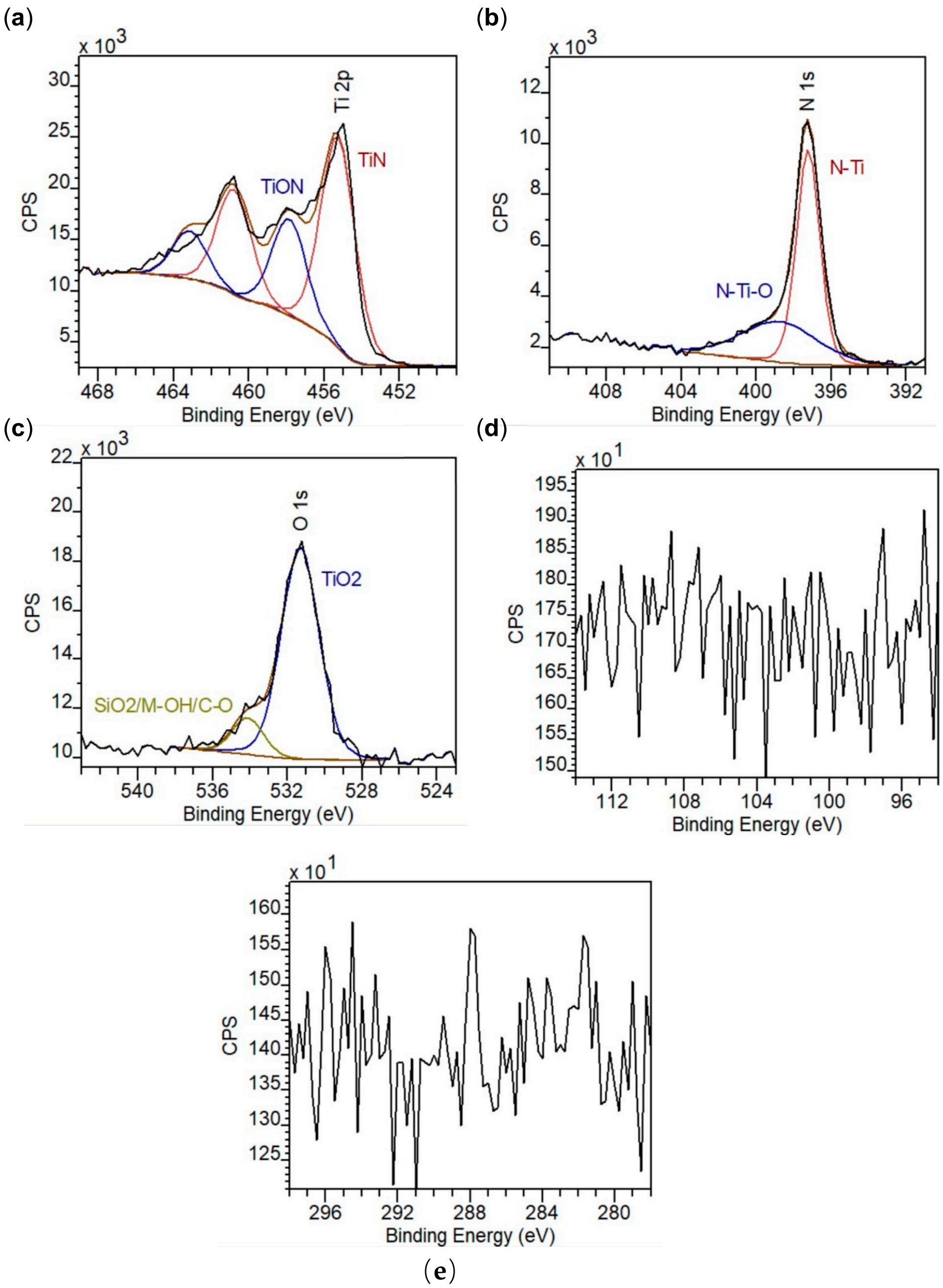

Figure S2. Structural characterization of 100 ALD cycles TiN on Si after H_2 post-deposition treatment using XPS (**a**) titanium 2p region, (**b**) nitrogen 1s region, (**c**) oxygen 1s region, (**d**) silicon 2p region, and (**e**) carbon 1s region.

© 2020 by the authors. Submitted for possible open access publication under the terms and conditions of the Creative Commons Attribution (CC BY) license (http://creativecommons.org/licenses/by/4.0/).
